# Children with autism show differences in the gut DNA virome compared to non-autistic children: a case control study

**DOI:** 10.1186/s12887-023-03981-8

**Published:** 2023-04-14

**Authors:** Aina Qu, Boyang Duan, Yue Wang, Zhenzhen Cui, Nuochen Zhang, De Wu

**Affiliations:** Pediatric Neurological Rehabilitation Center, The First Affiliated Hospital of Anhui Medical University, Hefei, China

**Keywords:** Autism spectrum disorder, Gut microbiome, Gut virome, Metagenome, Skunavirus

## Abstract

**Background:**

Several previous studies have identified a potential role that the gut microbiome can play in autism spectrum disorder (ASD) in children, but little is known about how variations in the virome may be involved in ASD. We aimed to understand the changes in the gut DNA virome of children with ASD.

**Methods:**

A case–control study was presented, in which 13 two-children families were observed while considering the age, mode of birth, history of antibiotic use, and vaccination history to minimize the influence of confounding factors. DNA viral metagenomic sequencing was successfully performed on stool samples from 11 children with ASD and 12 healthy non-ASD children. The basic composition and gene function of the participants' fecal DNA virome were detected and analyzed. Finally, the abundance and diversity of the DNA virome of children with ASD and their healthy siblings were compared.

**Results:**

The gut DNA virome in children aged 3–11 years was found to be dominated by the *Siphoviridae* family of *Caudovirales*. The proteins encoded by the DNA genes mainly carry out the functions of genetic information transmission and metabolism. Compared the gut DNA virome of ASD and healthy non-ASD children, their abundance of *Caudovirales* and *Petitvirales* both showed a significant negative correlation (*r* = -0.902, *P* < 0.01), there was no statistically significant difference in the relative abundance of viruses at the order and family levels, and a difference in the relative abundance at the genus level for *Skunavirus* (*Ζ* = -2.157, *P* = 0.031). Viral α diversity was reduced in children with ASD, but α diversity and β diversity did not differ statistically between groups.

**Conclusions:**

This study indicates that elevated *Skunavirus* abundance and decreased α diversity in the gut DNA virulence group of children with ASD, but no statistically significant difference in the change in alpha and beta diversity. This provides preliminary cumulative information on virological aspects of the relationship between the microbiome and ASD, and should benefit future multi-omics and large sample studies on the gut microbes in children with ASD.

**Supplementary Information:**

The online version contains supplementary material available at 10.1186/s12887-023-03981-8.

## Background

Autism spectrum disorder (ASD) is a neurological and developmental disorder, its main clinically characterized by impairments in social communication and interaction, behavioral problems, and diminished interests [1]. The global prevalence of ASD is about 1%, with males being 2–3 times more likely to be affected than females [2, 3]. On average, about 30% of affected children develop mental deterioration (i.e., loss of previously acquired skills) by 19.8 months of age [4], and more than 70% of patients with ASD have comorbidities such as gastrointestinal disorders, speech and language developmental disorders, epilepsy, depression, personality disorders, and schizophrenia [1–3], which result in significant caregiving, education, and medical costs to families and society [5]. Unfortunately, the etiology of the ASD is still not fully understood.

Several previous studies have identified a potential role of the gut microbiome in ASD, with pathogenic mechanisms being dynamic and bidirectional interactions between the gut flora and the brain along the microbiota-gut-brain axis [6]. During critical periods of development, children with ASD have an imbalanced gut microbiota that produces various metabolites and neuroactive substances that can activate the gut and vagus nerve endings to produce signals that are then transmitted to the brain or transferred to the brain through the disrupted intestinal barrier and the blood–brain barrier [7] or through the hypothalamic–pituitary–adrenal axis, where the immune system and the brain can interact abnormally [8]. The above abnormalities eventually cause neurodevelopmental disorders and behavioral abnormalities. Most of these results were obtained by detecting changes in the gut bacterial community, with very little analysis of other microbiota.

The gut microbiome includes bacteria, archaea, viruses, fungi and parasites [9], and there is an urgent necessity to develop a new field of investigation to gain insight into the variations (and possible effects of the variations) in the gut microbiome of children with ASD. As part of the gut microecology, the gut virome has nearly 10^15^ phages, 10 times more than bacterial cells and 100 times more than human cells [10], and phages can affect the gut microecology by altering the community composition of host bacteria [11]. Because the relative abundance of DNA viruses in human gut virome is above 80% [12], and it has been demonstrated that alterations in the intestinal virome, especially the DNA virome, may be important factors in diseases such as inflammatory bowel disease, colorectal cancer, diabetes, and Parkinson's disease. Therefore, the study of the gut DNA virome is an important and valuable approach to study the pathogenesis of diseases.

Here we conducted a case–control study to explore the differences in the gut DNA virome compared to non-autistic children, which might play a role in ASD. First, we recruited children with ASD from the same family to participate together with their healthy siblings, taking into account age, mode of birth, history of antibiotic use, and vaccination history to reduce the influence of confounding factors and to improve the accuracy of the test results. Stool specimens were then collected from children who met the recruitment criteria, and the basic composition and gene function of the participants' DNA virome were detected and analyzed by viral metagenomic sequencing. Finally, the contents of the DNA virome of children with ASD and their healthy siblings were compared to identify differences that may be related to the disease, to gain a better understanding of the relationship between intestinal microbes and ASD, and to increase the development of ASD etiology research in the field of microbial multi-omics.

## Methods

### Study population

**Fig. 1:**
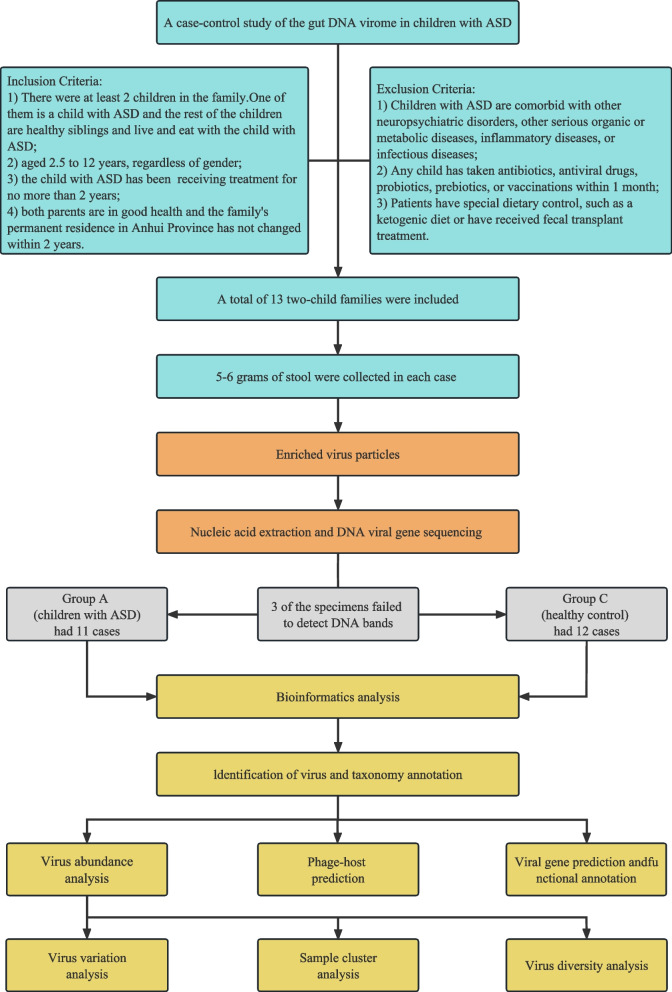
The schematic demonstration of the design of the presented study

In order to understand the changes in the gut DNA virome of children with ASD, this study was conducted at the First Affiliated Hospital of Anhui Medical University from November 2021 to May 2022 and as many children as possible meeting the criteria were recruited during this period. The recruitment criteria were as follows: (1) at least 2 children in the family: one child who met the diagnostic criteria for ASD as specified in the American Diagnostic and Statistical Manual of Mental Disorders, 5th edition [13] (Group A); the other child was a healthy sibling of the same parent as the child with ASD who lived and ate with the child with ASD (Group C) to reduce the influence of confounding factors; (2) aged 2.5 to 12 years, regardless of gender; (3) children with ASD had been treated for no more than 2 years; (4) both parents were in good health and the family's permanent residence in Anhui Province had not changed within 2 years. Exclusion criteria were as follows: (1) ASD combined with other neuropsychiatric diseases, other serious organic or metabolic diseases, inflammatory diseases, or infectious diseases; (2) the patient had taken antibiotics, antiviral drugs, probiotics, prebiotics, or vaccinations within 1 month; (3) the patient had special dietary control, such as a ketogenic diet or had received fecal transplantation treatment. The schematic demonstration of the design of the presented study is as follows (Fig. 1).

### Stool sample collection and storage

5–6 g of stool was collected from each child and immediately placed in a sterilized sampling tube containing preservation fluid (L005T, Shanghai Langfu Industry Co., Ltd, Shanghai, China), numbered (the authors could identify personal information by the number), and the samples were stored in a refrigerator at -80 °C until sent for testing. Samples were taken from two children from the same family at an interval of no more than three days.

### Enrichment of virus particles

Ultracentrifugation was used for the enrichment of viral particles. 5 g fecal sample was token to grinding and added 5 volumes of precooled sterile Stabilization Buffer, vortex for 5 min, after three rounds of freeze-thawing samples were centrifuged at 12,000 g for 5 min to remove the sediment, and cellular debris was removed using a 0.45 μm membrane. The supernatant was transferred to an ultracentrifuge tube containing 28% (w/W) sucrose, strictly leveled and centrifuged at 160,000 g for 2 h at 4 °C in a Himac CP 100WX ultracentrifuge (Hitachi, Tokyo, Japan). The supernatant was removed and the precipitate was resuspended in 200 μL Stabilization Buffer, and Enzyme Mix Buffer and Enzyme Mix were added proportionally. The enzyme Mix was incubated at 37 °C for 1 h; 2μL of termination solution was added, the enzyme mix and termination solution were mixed thoroughly, then the reaction was carried out at 65 °C for 10 min and centrifuged at 2000r for 5 min, and 200 ul of supernatant was stored at -20 °C.

### Nucleic acid extraction and DNA viral gene sequencing

DNA and RNA from the subjects’ feces were extracted using the Magen R6662-02 MagPure Viral DNA/RNA Mini LQ Kit, and whole genome amplification was performed using the Qiagen 150,054 REPLI-g Cell WGA & WTA Kit, followed by Thermo NanoDrop One, Life Technologies Qubit 4.0 and 1.5% agarose electrophoresis to ensure the quality of the amplified products. Samples that passed the quality control tests were sequenced using the NEB Next® Ultra II™ DNA Library Prep Kit for the Illumina platform (NEB, USA), and libraries were constructed. Libraries were then quality-checked and the samples were prepared in the machine according to the Illumina Novaseq 6000 platform User Guide and run in 150 bp paired-end mode in order to obtain raw reads for storage in FASTQ file format.

### Bioinformatics analysis

Low quality data obtained were removed from the original reads using the Trimmomatic (v0.36) software to obtain clean reads [14] and then compared with the ribosome database (Silva.132) and the host database using the software BWA (v0.7.17, default parameter: mem-k 30) [15]. After performing the comparison, results with a comparison length of less than 80% of the total length of the reads were filtered out and the corresponding host sequences were removed. Comparisons were then performed with the Virus-NT database, and comparison results with a comparison length less than 80% of the total length of the reads were also filtered out.

The contigs were obtained by assembling the de-hosted filtered reads using Megahit (v1.1.2, default parameters: –presets meta-large –min-contig-len 300) [16], and the clean reads were compared with the assembled results using BWA software. The utilization of the reads was calculated. Check V software [17] was used to predict the potential set of viral sequences in the assembled alleles, and then Virsorter2 software was used to identify the viruses in the initially assembled contigs again in order to further confirm the previous identification results [18], from which high-confidence sequences were selected to complement the Check V results.

After obtaining the set of potential viral contigs, VPF-Class software was used to compare the contigs in the viral protein family database (IMG/VR database), annotate the taxonomic information of viral contigs, and combine the annotated information of VPF-Class and Check V in the database to confirm the specific information of the viral contigs. Classification of virus sequences was performed. Also, host prediction of phages was performed based on the Baltimore relationship of protein sequences of viral signature genes in the VPF database [19]. The gene sequences of viral alleles were predicted using Prokka software (v1.13) to remove all allelic sequences with gene nucleic acid lengths below 200 bp and to evaluate the number and length of predicted genes [20]. Homology comparisons of gene sequence sets were performed using the Kyoto Encyclopedia of Genes and Genomes (KEGG) gene database using Diamond software with a threshold set to e-value ≤ 0.001, and the sequence with the lowest e value was selected for comparison of KEGG functional annotations [21, 22].

Viral sequences with genotype RNA were filtered out, and after decontamination, clean reads were compared with viral contigs using BWA software. Reads that were less than 80% of the total length of the reads were removed, the proportion of viral reads was counted, and the distribution of viral reads was counted based on the annotation results of the viral contigs. Finally, reads per kilobase per million mapped reads (RPKM) values were calculated for each virus contig for virus richness analysis, samples were clustered using the hclust function, and calculation of α diversity(Shannon index was used) and β diversity of samples based on the QIMME2 platform.

### Statistical analysis methods

SPSS 23.0 software, GraphPad Prism 9, and R were used for statistical analysis and graphing of the data in this experiment. The data quality and outlier detection were verified by the Grubb’s test.For comparison between two groups of data, the mean ± standard deviation was used for normally distributed continuous variables with Student's t-test; median and interquartile spacing were used for non-normally distributed continuous variables with Wilcox rank sum test; categorical variables were expressed as number of cases (percentage) with two-sided Fisher's exact test. *P* < 0.05 indicates a statistically significant difference.

## Results

### Clinical characteristics of the participating children

After a rigorous inclusion and exclusion process, children were included who were 3–11 years old and had no history of asphyxia, hyperbilirubinemia, cytomegalovirus infection, or rubella virus infection. Twenty-six stool specimens were collected from 13 Han Chinese families (See Supplemental Table 1). Three of the specimens failed to detect DNA bands, so the fecal gut DNA virome from 11 children with ASD (Group A) and 12 healthy siblings (Group C) was analyzed. Due to the small effect of hormones on the gut microbiology of children, which can be temporarily ignored [23], and because of the higher prevalence of ASD in males, no gender restriction was used in the recruitment of the study population and the two groups were not matched. Other clinical characteristics such as age, body mass index (BMI), mode of birth and postnatal feeding practices were similar between the two groups (Table 1).

**Table 1:** Basic clinical characteristics of the participants

Parameter	Group A (*n* = 11)	Group C (*n* = 12)	*P*-value
Female,n(%)	11(100%)	7(58.3%)	*P* = 0.037
Age (years)	3(3.0,6.0)	6(4.0,8.8)	*P* = 0.063
BMI(kg/m^2^)	17.63 ± 4.13	17.63 ± 2.22	*P* = 0.997
Vaginal delivery, n (%)	6(54.5%)	7(58.3%)	*P* = 1.000
Breastfeeding time, n (%)	7(63.6%)	8(66.7%)	*P* = 1.000
Complementary feeding (months)	6(5.0,6.0)	6(4.3,7.8)	*P* = 1.000
Gastrointestinal symptoms or food allergy	7(63.6%)	5(41.7%)	*P* = 0.414
Diet preferences(yes)	7(63.6%)	5(41.7%)	*P* = 0.414
Use anti-infective agents frequently(yes)	2(18.2%)	1(8.3%)	*P* = 0.590

Data are presented as mean ± standard deviation (SD), median with interquartile range (IQR), or n (%). The *p* values are based on the Student’s t test for variables expressed as mean ± SD, Wilcoxon rank-sum test for variables expressed as median (IQR) and two-sided Fisher’s exact test for variables expressed as percentages. Group A:children with ASD, Group C:healthy sibling group. *BMI *Body mass index

### Data overview of the viral metagenomic sequencing process

Viral metagenomic sequencing was performed on 23 stool specimens. First, quality control was performed on the preliminary raw reads data obtained. On average, 34,293,680 clean reads were obtained per sample, and 31,503,355 ± 9,530,239 filtered reads per sample after removing the host human sequences. Based on statistical analysis, the *p*-values between Group A and Group C were greater than 0.05 for most sequencing depths and were similar for both groups (See Supplemental Table 2 and Supplemental Fig. 1).

The average utilization rate of the reads was 94%, and the percentage of contigs that could be identified as host (Human) was already low, ranging from 0 to 2.52%. After removing the host contigs again, an average of 38,145 contigs per sample were obtained, and 76.6% of the contigs were within less than 1000 bp in length (See Supplemental Table 3 and Supplemental Fig. 2). Ultimately, 68,283 viral contigs were assembled from 23 children's stool samples for subsequent analysis.

### Basic composition of the gut DNA virome

#### The gut DNA virome of children with the vast majority being phages

**Fig. 2:**
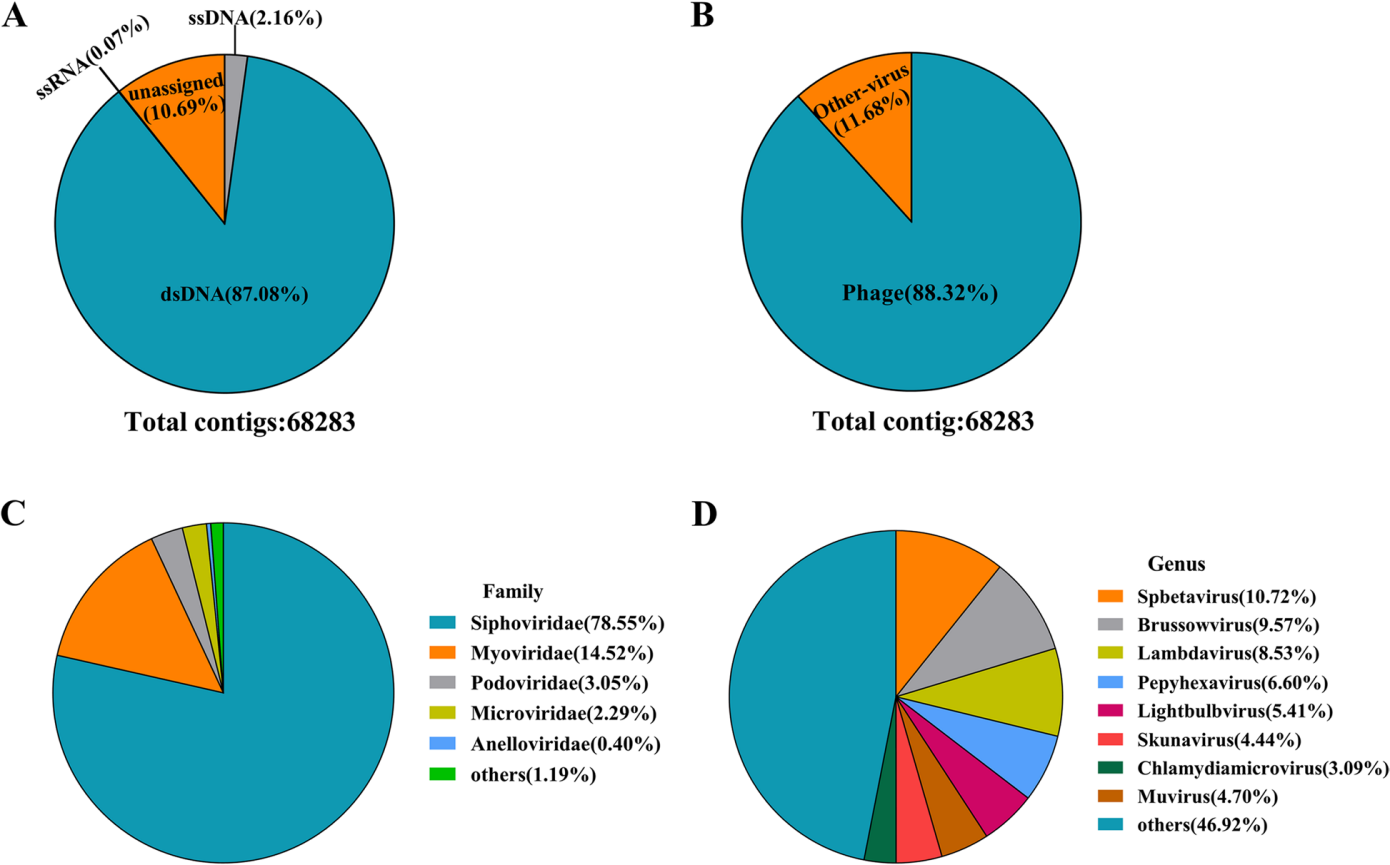
Virus contigs identification and taxonomic annotation results. **A** The proportion of virus contigs identified as DNA viruses. **B** The proportion of virus contigs identified as phage. **C** At the family level, virus classification annotated statistics. **D **At the genus level, virus classification annotated statistics

Sixty-eight thousand two hundred eighty-three viral contigs were identified and classified. Viral genome types that could be identified as dsDNA accounted for 87.08%, followed by 10.69% of contigs for which virus type could not be identified. 2.16% and 0.07% of viral genotypes were ssDNA and ssRNA, respectively, and the number of contigs identified as dsRNA was 0 (Fig. 2A). 88.32% of all viral contigs were identified as phages (Fig. 2B). The vast majority of the participants' gut DNA virome were phages.

#### The gut DNA virome is mostly composed of the *Siphoviridae* family of *Caudovirales*

Next, the contents of the identified gut DNA virome were biologically classified and were found to include 23 orders and 33 families. At the order level, 97.57% of the viruses were *Caudovirales,* followed by 1.52% that were *Petitvirales*, and a negligible proportion were other types of viruses (See Supplemental Table 4). At the family level, the *Siphoviridae*, the *Myoviridae*, and the *Podoviridae* under the order *Caudovirales*, which were in a dominant position, constituted the majority of the childhood gut virome, at 78.55%, 14.52%, and 3.05%, respectively. They were followed by *Microviridae* with 2.29% and *Anelloviridae* with 0.4%. (See Supplemental Table 4 and Fig. 2C). Most viruses could not be identified at the genus level. Among those that could be identified, *Spbetavirus* (10.72%), *Brussowvirus* (9.57%), and *Lambdavirus* (8.53%) were the top ranking three ranked viruses (See Supplemental Table 4 and Fig. 2D). In our study, most of the gut virome could only be identified at the order and family level, and the gut DNA virome of children aged 3–11 years was dominated by the *Siphoviridae* family of *Caudovirales*.

In addition, it was found that there are some specific viruses with very small numbers in the gut virome, such as *Herpesviridae*, which can cause disease, *Avipoxvirus*, a genus of *Poxviridae*, which can infect birds and grow in avian culture cells, *Ascoviridae*, which can infect invertebrates, *Chloroviridae*, a large virus that replicates in green algae, *Pandoravirus,* the largest known virus in the genome at present, and various others. Apparently, some of these viruses have no pathogenic effect in the intestinal tract of children.

#### Host prediction of enteric phages

**Fig. 3:**
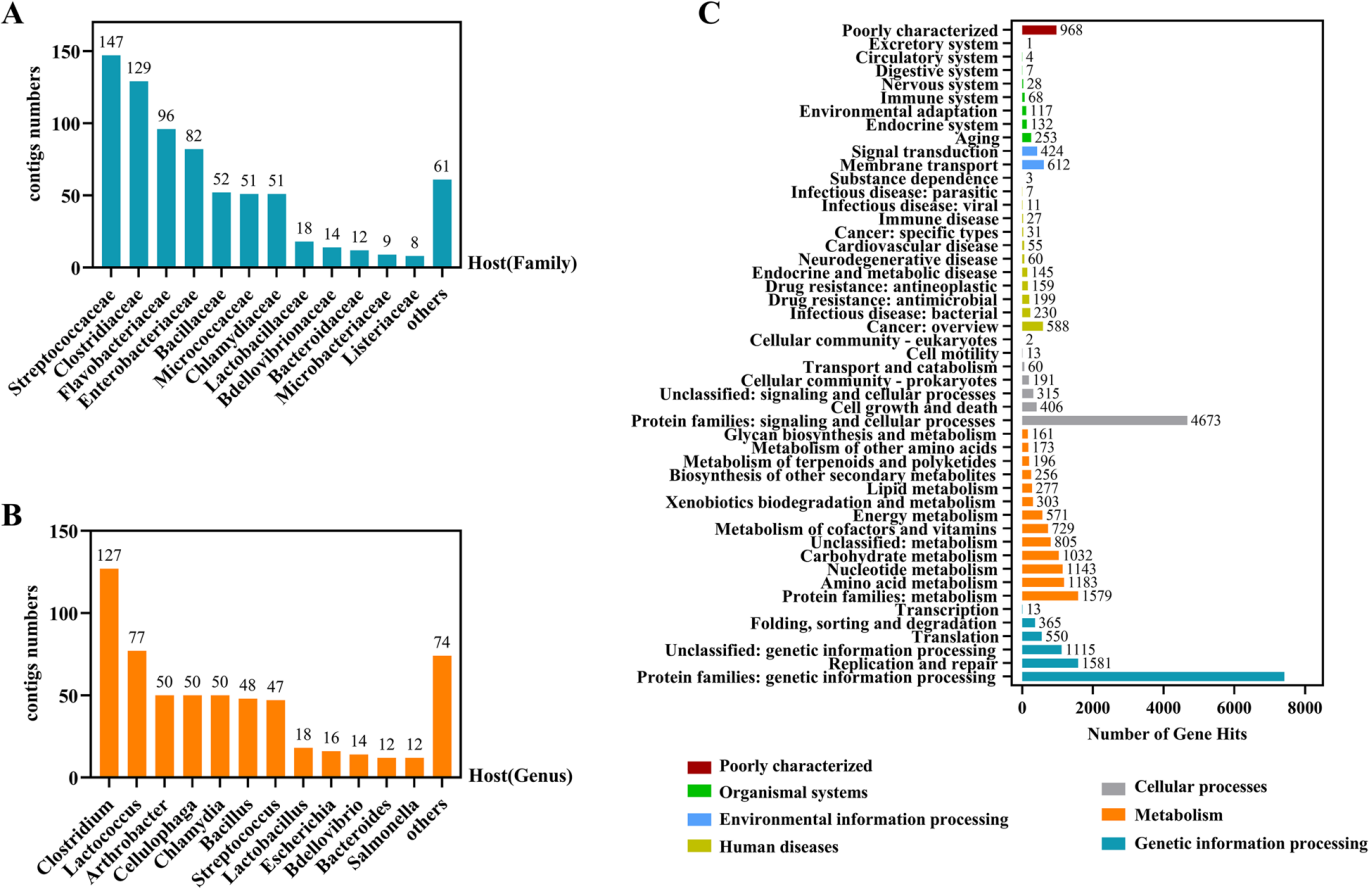
Functional analysis of genes and prediction of phage hosts. **A** Phage host prediction based on VPF-Class method(Family). **B** Phage host prediction based on VPF-Class method(Genus). **C **Gene functional analysis and number of gene hits according to KEGG classification(level 2)

In addition, we found it necessary to further investigate the hosts of phages that dominate the gut DNA virome and searched the VPF database to predict hosts at the domain, family, and genus levels. At the domain level, 99.6% of the hosts were bacteria and 0.4% were human eukaryotic cells. The most predominant hosts predicted at the family level were *Streptococcaceae* with 20.14%, followed by *Clostridiaceae* (17.67%), *Flavobacteriaceae* (13.15%), and *Enterobacteriaceae* (11.23%) (Fig. 3A). At the genus level, *Clostridium* (21.34%) was the predominant phage host, followed by *Lactococcus* (12.94%), *Arthrobacter* (8.4%), *Cellulophaga* (8.4%), and *Chlamydia* (8.4%) (Fig. 3B). Various other phage hosts were also identified, including pathogenic bacteria in some cases, such as *Staphylococcus, Acinetobacter, Enterococcus,* and *Haemophilus*, but they represented only a small percentage. The types of bacterial hosts predicted here for phages could inform future studies investigating phage-host interrelationships.

#### Gene function annotation of the gut DNA virome

To further understand the major gene functions of the childhood gut DNA virome, we homology-matched the gene sequences of viral contigs using the KEGG database and ranked them from largest to smallest according to the number of genes in each functional hit as genetic information processing, metabolism, cellular processes, human diseases, environmental information processing, and biological systems. It was.shown that the vast majority of protein families perform genetic information processing functions, followed by the functions of replication and repair involved in genetic information. Among the metabolic functions, amino acid metabolism, nucleotide metabolism, and sugar metabolism were predominant; among the cellular processes, the involvement of protein families in signal transduction and cellular processes was predominant; among human diseases, the number of genes related to tumors and bacterial infectious diseases was high; and among the functions of biological systems, those related to aging were predominant (Fig. 3C). In general, the proteins encoded by the genes in the childhood gut DNA virome mainly perform functions of genetic information transmission and metabolism.

### Abnormalities in the gut DNA virome of children with ASD

After obtaining a better understanding of the basic components of the gut DNA virome in children, we compared and analyzed differences in DNA virus abundance and diversity between children with ASD and their healthy siblings to detect changes in the gut DNA virome associated with ASD.

#### Alterations in the abundance of the gut DNA virome in children with ASD

By calculating the RPKM values for each viral contig and comparing the differences in relative abundance percentages greater than 1% at the order, family, and genus levels, we found that the abundance of *Skunaviruses* at the genus level differed between groups.

**Fig. 4:**
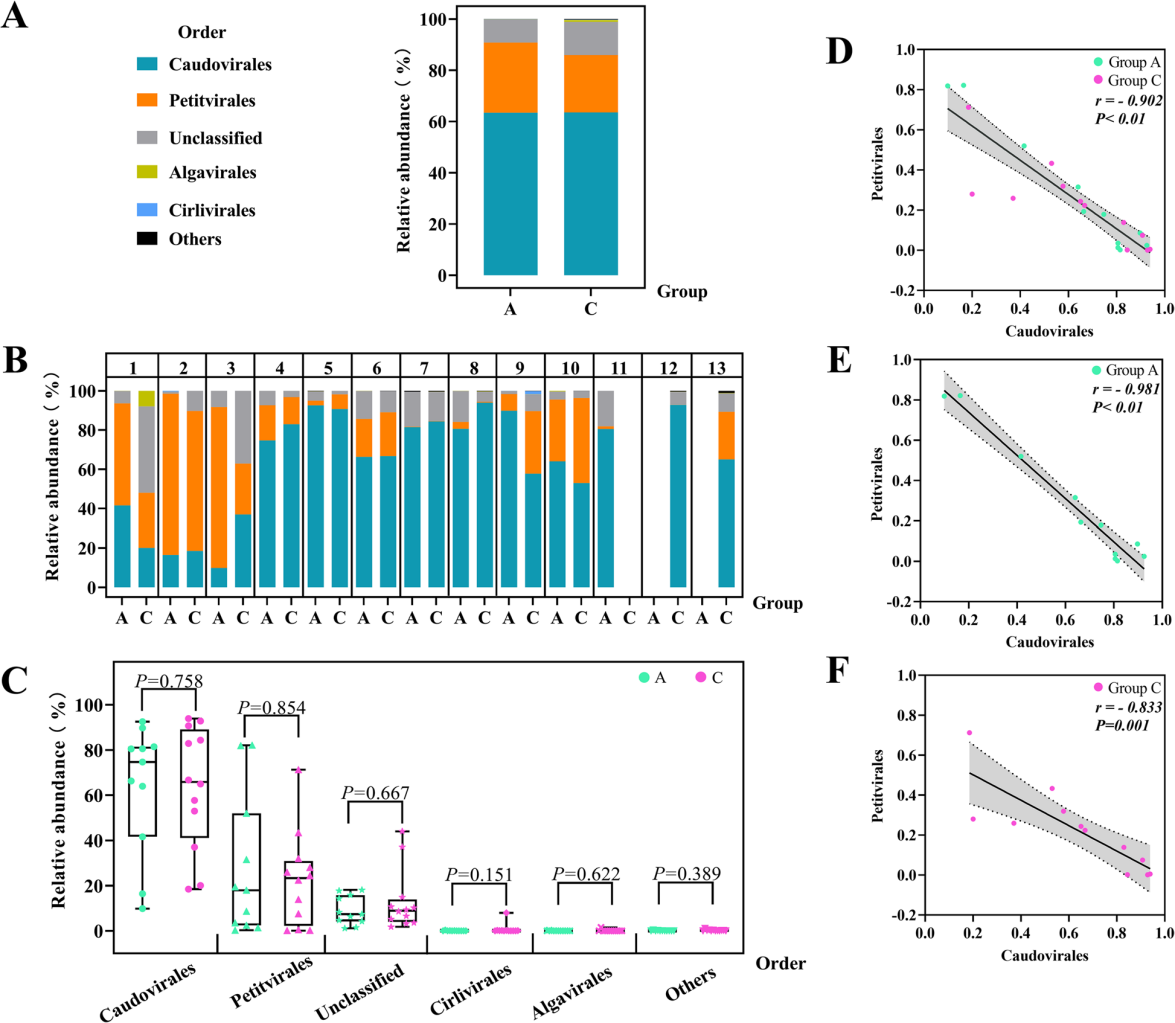
Relative abundance of viruses at the order level. **A** Relative abundance of gut DNA virome at the order level in the two groups. **B** At the order level, the relative abundance of viruses in each sample from the 13 households. **C** No significant difference between the two groups of viruses at the order level. **D** A negative correlation between *Caudovirales* and *Petitvirales* in ASD children and their healthy siblings. **E** A negative correlation between *Caudovirales* and *Petitvirales* in ASD patients. **F** A negative correlation between *Caudovirales* and *Petitvirales* in healthy siblings

First, at the order level, the gut DNA virome of children with ASD and their healthy siblings was dominated by *Caudovirales* and/or *Petitvirales* (Fig. 4A and B), but these viruses did not differ statistically significantly between the two groups (Fig. 4C). As for *Caudovirales* and *Petitvirales*, which were the top ranking two in relative abundances, they showed a negative correlation (*r* = -0.902, *P* < 0.01, Fig. 4D) with a correlation coefficient of 0.902, which indicates a very close correlation between them. This close negative correlation also persisted when groups A and C were observed separately (Fig. 4E and F).

**Fig. 5:**
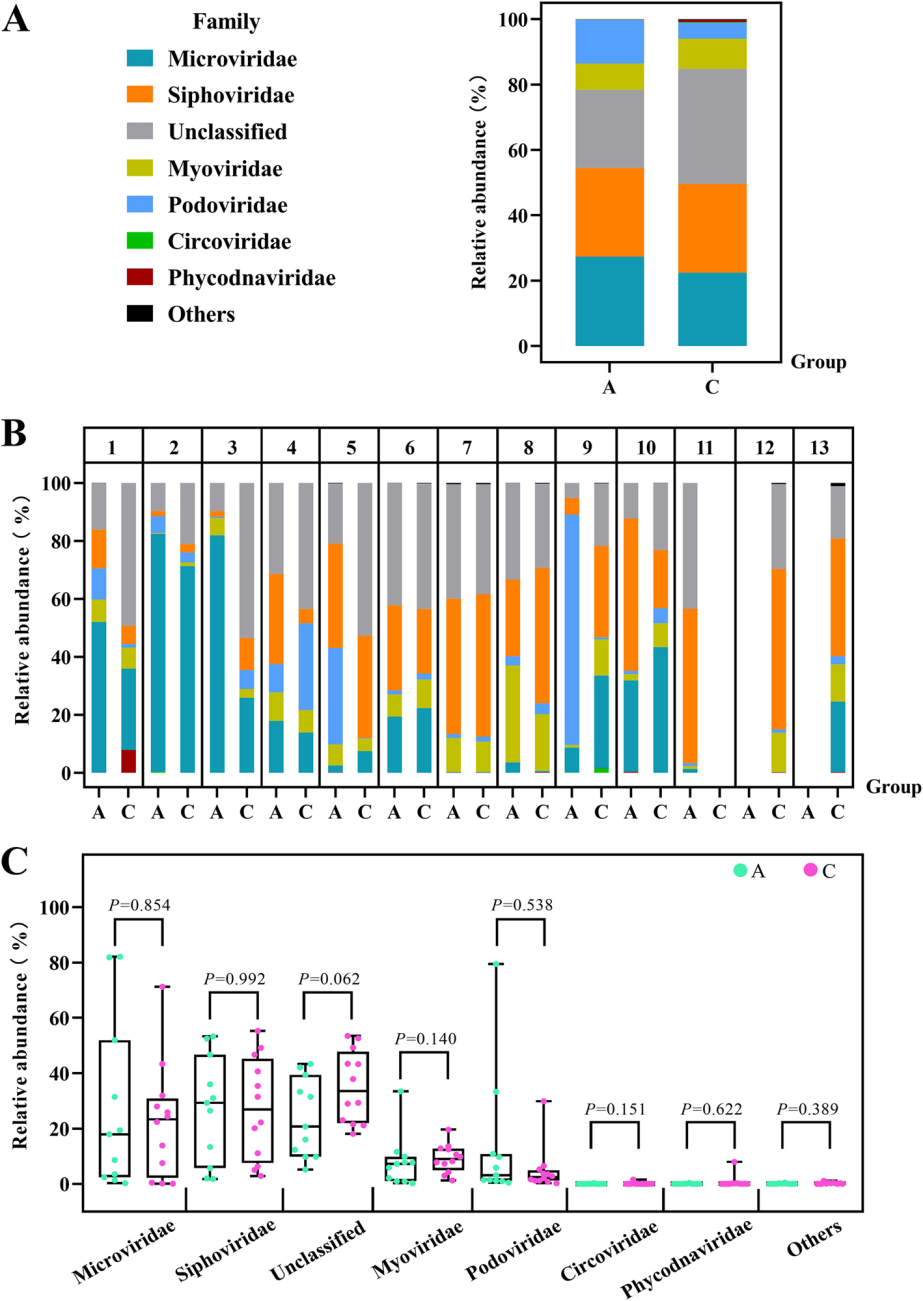
Relative abundance of viruses at the family level. **A** Relative abundance of gut DNA virome at the family level in the two groups. **B** At the family level, the relative abundance of viruses in each sample from the 13 households. **C** No significant difference between the two groups of viruses at the order level

At the family level, there was no significant difference in the abundance of viruses between the two groups. At this level, differing from the percentages of each virus in contigs, the relatively high abundance is in the group of viruses whose classification cannot be determined by current detection methods (about 29.57%), followed by *Siphoviridae* (27.15%), *Microviridae* (24.89%), *Podoviridae* (9.16%), *Myoviridae* (8.6%), etc. Among the identifiable viruses, the difference in the relative abundance of viruses between ASD children and healthy non-ASD children was approximately equal for *Siphoviridae* ( 27.11% versus 27.19%), showing the increased viral abundance of *Microviridae* (27.36% versus 22.43%) and *Podoviridae* (13.43% versus 4.89%), and showed a decreased abundance of *Myoviridae* (7.98% versus 9.22%) (Fig. 5A and B), but these differences were not statistically significant (Fig. 5C).

**Fig. 6:**
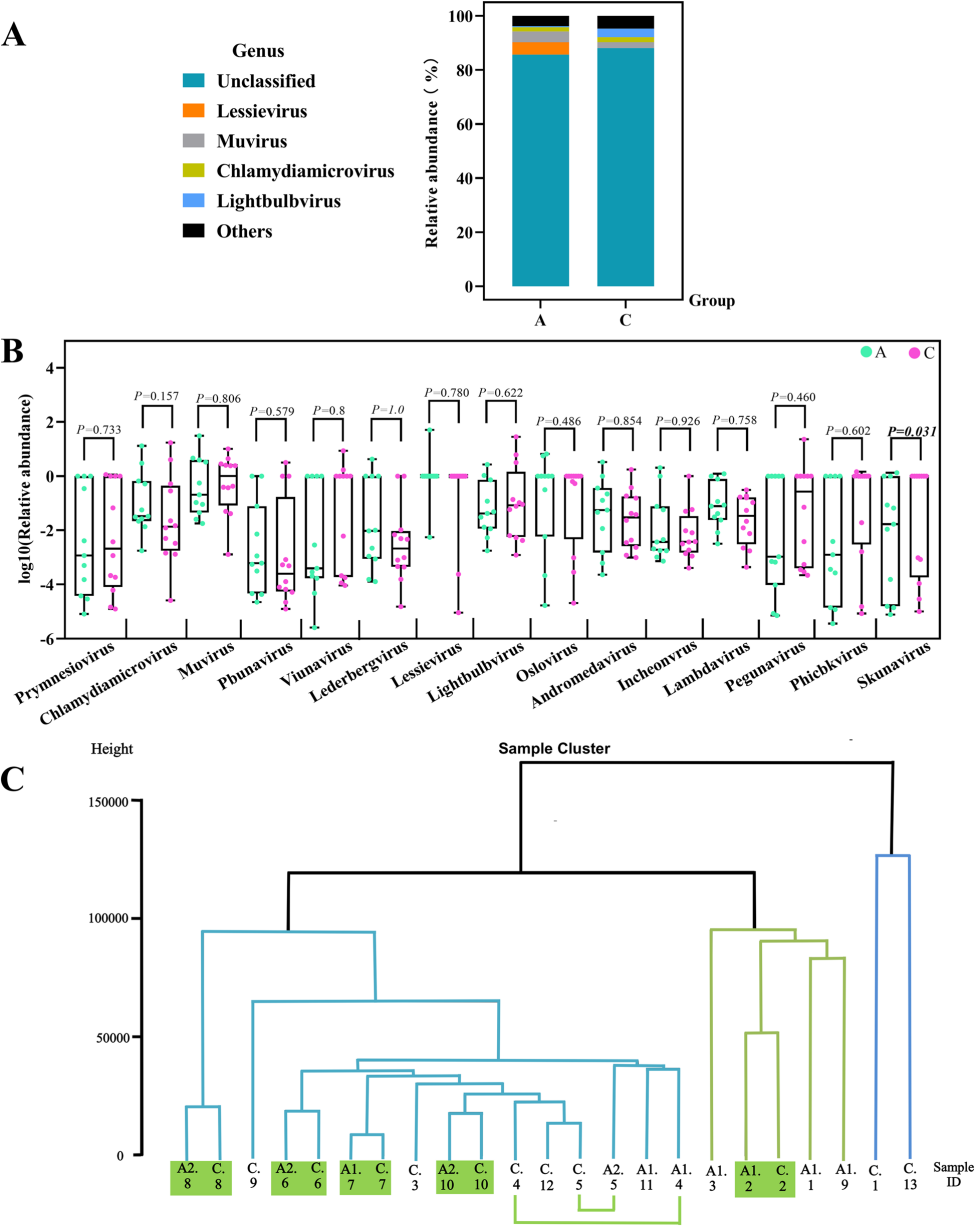
Relative abundance of viruses at the genus level and the dendrogram. **A** Relative abundance of gut DNA virome at the genus level in the two groups. **B** *Skunavirus* has a difference between the two groups. **C** Using the hclust function in the stats package in the R program and plotting as a dendritic clustering graph, with the height of the branch representing the distance between samples, and the horizontal line at the end of the branch representing a high degree of sample similarity and aggregation

Finally, at the genus level, a statistically significant difference in *Skunavirus* was shown between the two groups. To rank whether this difference was influenced by age, Pearson correlation analysis was performed between age and relative abundance of *Skunavirus* in 23 children and *P* = 0.297 indicated no correlation(See Supplemental Table 1 and 5). Limited by the current virus detection technology and thus a limited level of awareness of the virome, about 86.93%, on average, of the viruses in each sample could not be categorized at the genus level. As a result, the abundance of the viruses that could be identified was low, and Fig. 6A shows the distribution of viruses with relative abundance in the top 4 among the two groups of children, with the rest of the virome classified as "others". When the differences in the relative abundance of viruses greater than 1% among children with ASD and their healthy non-ASD siblings were compared, a statistically significant difference was found between the two groups for *Skunavirus* with low relative abundance (*Ζ* = -2.157, *P* = 0.031), while the top 4 viruses with slightly higher viral abundance, *Lessievirus, Muvirus, Lightbulbvirus*, and *Chlamydiamicrovirus,* showed no significant statistical difference between the two groups (Fig. 6B).

In addition, during the virus richness analysis, it was found that the similarity of the gut virome in two children within the same households were higher on average than that of children from other households (Fig. 4B and 5B.) Therefore, the hclust function in the stats package in the R program was used to compare the similarity and differences between samples and plotted as a dendritic clustering plot, with the height of the branches representing the distance between samples, and the horizontal line at the end of the branch representing a high degree of sample similarity and aggregation. As shown in Fig. 6C, among the 10 groups of households in pairs, households 8, 6, 7, 10, and 2 are clustered close to each other between two individuals, households 4 and 5 also showed high similarity between the two children, and households 1, 3, and 9 showed low similarity between the two children's viromes.

#### Characteristics of intestinal DNA virome diversity in children with ASD

**Fig. 7:**
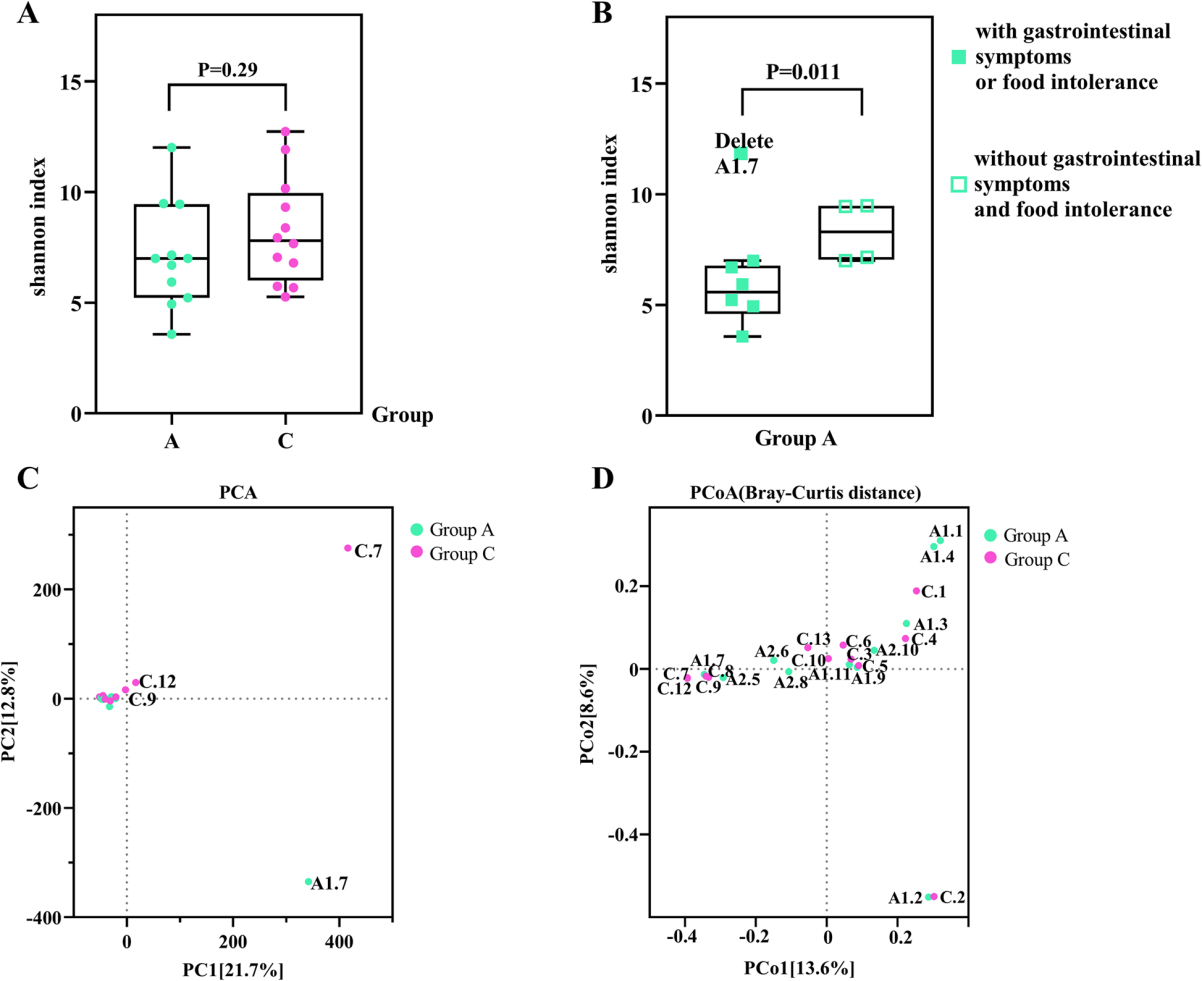
Gut DNA virome diversity. **A** Shannon index of the two groups of virus diversity indicators are not statistically different. **B** After removing a limiting value in children with ASD(Sample ID: A1.7), there was a statistical difference between those with gastrointestinal symptoms or food intolerance and those without symptoms on the Shannon index. The data quality and outlier detection were verified by the Grubb’s test. **C** Principal component analysis(PCA). **D** Principal coordinates analysis (PCoA) based on the Bray–Curtis distance. Each point in the C) and D) plots represents a sample, and the point-to-point spatial distances indicate the magnitude of differences in species community structure; principal component 1 (PC1 or PCo1) on the X-axis and principal component 2 (PC2 or PCo2) on the Y-axis represent the two major eigenvalues that contribute to differences between samples, followed by percentages that indicate the variance contribution of the two principal components to sample differences

As for the diversity of the enteric virome, the differences between children with ASD and their healthy non-ASD siblings did not show significance. We used the Shannon index to express the α diversity of the virome as a comprehensive analysis of the richness and homogeneity of the species in individual samples. It turned out that the α diversity in ASD children was decreased, but this decrease was not statistically significant (Fig. 7A). To identify possible differences in α diversity between individuals, subgroup statistical analyses were performed in combination with the evaluation of other clinical characteristics of the children. Comparing the Shannon index in children with gastrointestinal symptoms or food intolerance with those without symptoms in groups A and C, respectively, there was a statistically significant difference shown between subgroups in group A (*Ζ* = -2.558, *P* = 0.011) after removing one extreme value (Sample Id: A1.7, Fig. 7B, outlier detection was verified by the Grubb’s test), and no statistically significant difference was shown between subgroups in group C (*P* = 0.354). Within group C, no significant correlation was shown between gender and α-diversity (*P* = 0.371).

Next, the β-diversity of the gut virome was illustrated by a principal component analysis (PCA) and a principal coordinates analysis (PCoA) based on the Bray–Curtis distance. It was found that no two clear subgroups were formed between the samples and the gut virome of ASD children and healthy siblings were processed by dimensionality reduction, and that the distance between individuals mapped at each point in the graph was close (in the cases of some individuals, it almost overlapped), which indicates that the virome communities of the two groups of children did not differ significantly in terms of β-diversity (Fig. 7C and D).

## Discussion

To our knowledge, this is the first report that specifically explores whether there are differences in the gut DNA virome in children with ASD compared to healthy non-ASD children. The age range of the children involved in this study was 3–11 years, and microbiological analysis of a total of 23 samples revealed that the gut DNA virome of the participants was dominated by the *Siphoviridae* family of *Caudovirales*. The results showed that the abundance of *Caudovirales* was significantly and negatively correlated with that of *Petitvirales*, the relative abundance of gut DNA virome in children with ASD compared with their healthy siblings was not significantly different at the order and family level, however, there were differences at the genus level for *Skunavirus.* α diversity was decreased in children with ASD, but there was no significant statistical difference in viral α diversity and β diversity between the two groups.

There was no statistically significant difference in viral abundance between children with ASD and healthy non-ASD children by orders of magnitude. *Caudovirales* and *Petitvirales* were the top ranking two, and both viruses were strongly negatively correlated within each group, which indicates that viruses of high abundance naturally restrict viruses of low abundance [24], with the dominant virus dominating ecological functions in the gut. It has recently been shown that *Caudovirales*, in particular the *Siphoviridae* family, are associated with better executive function and memory in the human gut, while *Microviridae* is associated with impaired executive function [25]. As demonstrated by our results, although *Microviridae* abundance was elevated in children with ASD, it did not cause statistically significant differences compared to normal children, while *Caudovirales* and *Siphoviridae* abundance in the intestine did not change significantly, in children who continued to perform their physiological functions. Demonstrating that the executive function and memory abilities of children with ASD were not severely disrupted by the disease, and they were able to perform rehabilitation tasks well and complete various memory challenges.

At the genus level, intergroup differences in *Skunavirus* viral abundance were observed between ASD children and healthy non-ASD children. *Skunavirus* belongs to the *Caudovirales, Siphoviridae* family, is generally present in raw cow's milk, and cannot be eliminated by pasteurization [26]. As a lysogenic phage, *Skunavirus* can cause slow milk fermentation and poor quality of fermented products by invading and lysing *Lactococcus lactis*. Recently, a large amount of research on this process has been done in the field of food processing [27, 28]. Because the relative abundance of *Skunavirus* in the intestine of children in this experiment was about 1.5% (See Supplemental Table 5), and no articles describing *Skunavirus* and its activity in the human intestine were retrieved, we can only speculate about the results of this study: the possibility that *Skunavirus* abundance was age-related was also excluded, perhaps the higher amount of *Skunavirus* in milk or milk products consumed during early childhood has caused an increased abundance of *Skunavirus* in the intestine compared to normal children, thus playing a potential role in the development of ASD in children. Future sample size expansion, multiple sampling, and multiple replicate experiments are needed to test the conjecture.

In the diversity analysis of the gut DNA virome, no significant differences in α diversity and β diversity were shown between children with ASD and their healthy non-ASD siblings. Since the human gut is overwhelmingly populated by mild phages [29], the more similar the bacterial group of an individual, the more similar the viral group, and the diversity of bacteria and viruses are correlated [30]. Therefore, our findings are similar to those of Son et al. in their analysis of the relationship between intestinal flora and ASD. In their study, the gut flora 16S rRNA in 59 children with ASD and 44 neurodevelopmentally normal siblings were sequenced and no significant differences in gut flora α or β diversity between the two groups of children were found [31]. To further confirm this conclusion, Yap et al. performed a metagenomic analysis of the gut flora on 99 children with ASD, 51 healthy siblings of these ASD children, and 97 unrelated healthy non-ASD children, in which they found no association between gut flora and ASD, and concluded that the altered diversity of gut flora in children with ASD was caused by dietary preferences [32]. All of our experiments fully considered various confounding factors that can affect gut microbes, and the comparison between siblings allowed for adequate matching to assure that genetic, ethnic, residential, environmental, and dietary factors, as well as age, birth mode, postnatal feeding mode, body mass index, gastrointestinal dysfunction, and dietary preference were comparable between the two groups in this experiment. Additionally, excluding the effects that the use of drugs such as antibiotics and vaccines could have had on the results increased the accuracy in demonstrating the true distribution of viral communities in the intestinal tract of diseased and healthy children.

Another phenomenon worth discussing in the analysis of α diversity is that in children with ASD, α diversity was significantly lower in children with gastrointestinal symptoms compared which was not the case in healthy non-ASD children. Given the potential trend in the viral group of digestive dysfunction in children [33, 34], it can be assumed that in healthy children, the viral group recovers from gastrointestinal symptoms by self-regulation, thus making the difference insignificant. However, in children with ASD, when the intestinal barrier has been disrupted or the composition of the gut virome itself has been significantly altered, the α diversity of the virome decreases once gastrointestinal dysfunction is present. In addition, dietary behaviors and dietary patterns can affect the gut microbes in ASD children [32, 35], and it is probable that the degree of dietary preference in children with ASD in the diseased state is more severe than in normal children. It can also be difficult to regulate dietary behaviors and thus reduce the gastrointestinal burden and improve gastrointestinal function in ASD patients, which can result in altered gut virome diversity. However, Gondalia et al. found no significant differences in gut flora between ASD children with and without gastrointestinal dysfunction [36]. One possible reason for the difference in findings could be body mass index. It has been shown that the intestinal flora and viral communities are altered in obese individuals [37, 38], and our analysis removed a limiting value that possibly influenced the conclusion that the clinical characteristic of the children represented by this significantly different value was obesity (body mass index of 26.63 kg/m^2^), whereas Gondalia did not mention the body mass index of the participants in his article. It is also possible that our experiment was too homogeneous, as the intestinal symptom of these children was constipation (See Supplemental Table 1). More studies on the virome are merited in the future to determine the relationships between them.

The main limitations of this experiment are the following: (1) Since this is a preliminary exploration of the gut virome in children with ASD and is costly, we collected a small sample size for DNA virome testing. (2) Only 1 sampling analysis was performed, and it was not possible to detect any abnormalities in the fluctuations of the gut virome during the growth and development of the children. (3) We did not record the dietary structure of the children in detail, their preferred foods, or the severity of the disease in order to ascertain whether there would be different distributions of the gut virome in these different conditions.

## Conclusions

In conclusion, the findings suggest some alterations in the structure of the intestinal DNA virome in children with ASD, elevated *Skunavirus* abundance and decreased α diversity in the gut DNA virulence group of children with ASD, but no statistically significant difference in the change in alpha and beta diversity. Our study intends to fill a gap in understanding regarding the presence or absence of alterations in the gut virome in children with ASD, and indicate that other microbiomes may also be associated with ASD. Future studies should include larger cohorts, a longitudinal collection of samples, and multi-omic studies of microorganisms such as bacteria, viruses, and fungi, combined with animal experiments for further studies of pathogenic mechanisms.

## Supplementary Information

**Additional file 1.**

**Additional file 2.**

## Data Availability

The raw sequence data reported in this paper have been deposited in the Genome Sequence Archive (Genomics, Proteomics & Bioinformatics 2021) in National Genomics Data Center (Nucleic Acids Res 2022), China National Center for Bioinformation / Beijing Institute of Genomics, Chinese Academy of Sciences (GSA: CRA009871) that are publicly accessible at https://ngdc.cncb.ac.cn/gsa. Editors and reviewers can view the database information through a shared link before the article is published: https://ngdc.cncb.ac.cn/gsa/s/Hy4Mpu10.

## Abbreviations

ASD: Autism spectrum disorder
KEGG: Kyoto Encyclopedia of Genes and Genomes
RPKM: Reads per kilobase per million mapped reads
BMI: Body mass index
