# Supplementary material for: Children with autism show differences in the gut DNA virome compared to non-autistic children: a case control study
Source: BMC Pediatr. 2023 Apr 14;23:174. doi: 10.1186/s12887-023-03981-8 (PMC10105470; doi:10.1186/s12887-023-03981-8)
Supplement: Supplementary file 1 — Additional file 1. [file 12887_2023_3981_MOESM1_ESM.zip › Supplemental Figure 1.doc]

**Supplemental Figure 1.** Virus sequences detected in ASD and healthy siblings samples. Plots containing the total number of sequences(raw-reads)(A), quality sequences(clean-reads)(B), and quality-filtered sequences(filtered-reads in which human sequences were removed) (C) are shown. Error bars indicate the median and interquartile range. The number of sequences per sample is shown as points. There were no statistically significant differences between the two groups.
