# Supplementary material for: Children with autism show differences in the gut DNA virome compared to non-autistic children: a case control study
Source: BMC Pediatr. 2023 Apr 14;23:174. doi: 10.1186/s12887-023-03981-8 (PMC10105470; doi:10.1186/s12887-023-03981-8)
Supplement: Supplementary file 1 — Additional file 1. [file 12887_2023_3981_MOESM1_ESM.zip › Supplemental Figure 2.doc]

**Supplemental Figure 2.** The number of contigs under different contig lengths per sample.
