# Supplementary material for: Children with autism show differences in the gut DNA virome compared to non-autistic children: a case control study
Source: BMC Pediatr. 2023 Apr 14;23:174. doi: 10.1186/s12887-023-03981-8 (PMC10105470; doi:10.1186/s12887-023-03981-8)
Supplement: Supplementary file 2 — Additional file 2. [file 12887_2023_3981_MOESM2_ESM.zip › 12887_2023_3981_MOESM2_ESM/Supplemental Table 1.Baseline characteristics of all the children cohort.doc]

| **Sample ID** | **Gastrointestinal**  **symptoms or food allergy** | **Long-term residence in Anhui Province** | **Gender** | **Age**  **(years)** | **BMI**  **(kg/m^2^)** | **Mode of**  **birth** | **Feeding mode**  **after birth** | **Time to add complementary food(months)** | **Diet preferences** | **Use anti-infective agents frequently** |
| --- | --- | --- | --- | --- | --- | --- | --- | --- | --- | --- |
| **A1.1** | Constipation  frequently | Hefei | Male | 3 | 17.44 | Vaginal delivery | Mixed feeding | 6 | Fried foods  Puffed foods  Carbonated drinks | No |
| **C.1** | None | Hefei | Female | 8 | 17.84 | Vaginal delivery | Mixed feeding | 6 | Desserts  Puffed foods  Carbonated drinks | No |
| **A1.2** | Constipation  frequently | Huainan | Male | 6 | 14.36 | Cesarean  section | Formula feeding | 24 | Desserts  Fried foods  Puffed foods  Carbonated drinks | Less than 20 days in a year |
| **C.2** | None | Huainan | Male | 6 | 14.36 | Cesarean  section | Formula feeding | 24 | None | No |
| **A1.3** | Food allergy | Hefei | Male | 5 | 14.24 | Vaginal delivery | Breastfeeding | 6 | None | No |
| **C.3** | None | Hefei | Female | 8 | 15.28 | Vaginal delivery | Breastfeeding | 6 | None | No |
| **A1.4** | Constipation  frequently | Hefei | Male | 3 | 15.42 | Vaginal delivery | Mixed feeding | 6 | Meat  Nuts   Dried fruits  Carbonated drinks | No |
| **C.4** | Constipation  frequently | Hefei | Female | 11 | 21.30 | Vaginal delivery | Breastfeeding | 4 | Meat  Desserts  Puffed foods  Nuts  Dried fruits | No |
| **A2.5** | None | Anqing | Male | 3 | 19.00 | Vaginal delivery | Breastfeeding | 6 | None | No |
| **C.5** | None | Anqing | Male | 10 | 20.76 | Vaginal delivery | Breastfeeding | 6 | None | No |
| **A2.6** | None | Hefei | Male | 7 | 17.56 | Vaginal delivery | Breastfeeding | 5 | Meat  Fried foods  Carbonated drinks | No |
| **C.6** | None | Hefei | Female | 3 | 17.00 | Vaginal delivery | Breastfeeding | 4 | None | No |
| **A1.7** | Food allergy | Hefei | Male | 7 | 26.63 | Cesarean  section | Breastfeeding | 5 | Meat  Desserts  Milk products  [Curing food](javascript:;) | No |
| **C.7** | Constipation  frequently | Hefei | Male | 4 | 16.53 | Cesarean  section | Breastfeeding | 5 | Meat  Desserts  Milk products  [Vegetable](javascript:;)s  Fruits | No |
| **A2.8** | None | Hefei | Male | 3 | 19.53 | Vaginal delivery | Breastfeeding | 8 | Meat  Desserts  Milk products  [Bean products](javascript:;)  Puffed foods | No |
| **C.8** | None | Hefei | Female | 5 | 17.00 | Vaginal delivery | Breastfeeding | 8 | Desserts  [Vegetable](javascript:;)s  Fruits  Milk products  [Bean products](javascript:;)  Puffed foods | No |
| **A1.9** | Food allergy | Hefei | Male | 3 | 22.50 | Cesarean  section | Breastfeeding | 4 | Desserts | No |
| **C.9** | None | Hefei | Male | 6 | 20.76 | Cesarean  section | Breastfeeding | 4 | None | No |
| **A2.10** | None | Hefei | Male | 6 | 13.00 | Cesarean  section | mixed feeding | 6 | None | No |
| **C.10** | Constipation  frequently | Hefei | Male | 3 | 15.98 | Cesarean section | mixed feeding | 6 | None | No |
| **A1.11** | Food allergy | Hefei | Male | 3 | 14.20 | Cesarean  section | Breastfeeding | 6 | Meat  Desserts  Milk products  Nuts  Dried fruits | Less than 20 days in a year |
| **C.11*** | None | Hefei | Female | 6 | 14.58 | Cesarean  section | Breastfeeding | 6 | Desserts  Milk products  Fried foods  Puffed foods | Less than 20 days in a year |
| **A2.12*** | None | Hefei | Female | 4 | 15.39 | Vaginal delivery | Mixed feeding | 6 | Meat  Milk products  Puffed foods | Less than 20 days in a year |
| **C.12** | Food allergy | Hefei | Male | 9 | 17.12 | Vaginal birth | Breastfeeding | 7 | Meat  Desserts  [Bean products](javascript:;) | No less than 20 days in a year |
| **A2.13*** | None | Hefei | Male | 6 | 15.80 | Cesarean  section | Formula feeding | 6 | Desserts  [Bean products](javascript:;)  Carbonated drinks | No |
| **C.13** | Food allergy | Hefei | Male | 4 | 17.64 | Cesarean  section | Mixed feeding | 8 | None | No |

Table S1.Baseline characteristics of all the children cohort.

A1: Autistic children with gastrointestinal symptoms or food allergy;

A2: Autistic children without gastrointestinal symptoms and food allergies;

*: The stool samples of these children failed to detect DNA bands and were not included in statistical analysis and sequencing;

BMI: Body mass index.
